# Two Cases of Gunshot Wounds: One of the Axilla, in Which the Subclavian Artery Was Tied. The Other, of the Popliteal Space, in Which the Femoral Artery Was Tied in Scarpa’s Triangle, with Secondary Amputation of Thigh Ten Days Later

**Published:** 1897-03

**Authors:** W. H. Hudson

**Affiliations:** LaFayette, Ala.


### TWO CASES OF GUNSHOT WOUNDS: ONE OF THE AXILLA, IN WHICH THE SUBCLAVIAN ARTERY WAS TIED. THE OTHER, OF THE POPLITEAL SPACE, IN WHICH THE FEMORAL ARTERY WAS TIED IN SCARPA'S TRIANGLE, WITH SECONDARY AMPUTATION OF THIGH TEN DAYS LATER.

By W. H. HUDSON, M.D ,

LA FAYETTE, ALA.

CASE 1. W. I., white, a healthy lad, aged fourteen years, was out hunting. In climbing over a high fence, pulling his gun after him by the muzzle, the gun discharged, the whole load entering his left axillary space. The charge was of bird shot, and a moderately large one. The wound bled profusely at the time, but a physician was soon obtained, who stopped the bleeding by packing the wound with cotton and muriated tincture of iron. The arm was bound to the side and there was no recurrence of hemorrhage until the sixth or seventh day, when a profuse hemorrhage occurred. The bleeding was stopped as before.

During the five weeks following there were five secondary hemorrhages, the wound being treated each time the same, packed with cotton and muriated tincture of iron.

During the sixth secondary hemorrhage I was telegraphed for, and on reaching the patient's bedside found the hemorrhage again under control. The boy was very pale from loss of blood, excited, and constantly beseeching his attendants not to allow him to bleed to death. In such a case, at the present time, I would immediately inject a saline solution, either subcutaneously or into the veins. In this case, however, this was not done.

From his general condition I thought my treatment well-nigh useless; but watched him during the night and had occasionally administered a little strychnia or brandy.

In the morning he had reacted somewhat, and I determined to pursue some course with the purpose of preventing the recurrence of the hemorrhage. The wound in the axilla had not healed to any extent, was considerably stained with the iron, but, nevertheless, was in fair condition. The hemorrhage had evidently come from the axillary artery, and the axillary vein was also, very probably, injured, as were the nerves.

I thought the most surgical thing to do was to amputate the arm at the shoulder joint. I feared that such an operation might be fatal. The next thing was to open the wound and ligate the bleeding vessels. This I did not think advisable, so I decided to ligate the subclavian artery in continuity above the clavicle, and trust to iodoform gauze packing, and cleanliness, to control the bleeding from the axillary wound.

Under ether narcosis the artery was tied at the place of election above the clavicle. A medium-sized braided silk ligature boiled in carbolic acid water was used. In tying the artery I attempted to avoid rupturing any of the coats.

The operation was done under thorough antiseptic and aseptic precautions. And allow me to say here that there is hardly a farm-house from one end of this country to another that is not prepared to afford facilities for first-class surgical results. During the last few years I have in such houses removed intra- and extra-dural blood-clots, brain tumors, and done laparotomies for gunshot wounds, and various other surgical operations with such results as are called in our leading hospitals, with their expensive operating-rooms, ideal.

One thing very noticeable during the operation was the almost total lack of bleeding; the tissues were well-nigh exsanguinated, more so than I have ever seen before or since in a living human being. It is a matter of regret that I was not prepared to estimate hemoglobin or to make a count of the blood corpuscles.

The case was not seen by me after the operation for twenty days. The operation-wound was then thoroughly healed, and the axillary wound was about filled in. I was informed that the recovery had been free from complications.

CASE 2. H. S., aged twenty-three years; mulatto; very strong and muscular; in resisting an officer was shot through the leg, the ball entering the center of the popliteal space, ranging downward and making its exit between the tibia and fibula, high up. The wounds did not bleed profusely at the time, but was seen by a physician a few hours later who cleansed the wounds and packed them with iodoform powder and applied a bandage.

The patient was placed in jail and the physician who attended him discharged him as cured about the tenth day after the shooting.

On the night of the fifteenth day the patient suffered intense pain in his leg, especially in the back part of the leg, which swelled and became very hard. The jail physician again attended him and asked me to see the case with him on the seventeenth day. I had the man removed to my office for better light, and for an operation, if one should be necessary.

He had been suffering intense pain, and had not slept for two previous nights. He did not appear to have fever, though his temperature was not taken; pulse not sufficiently rapid to attract especial attention; the leg was much enlarged, the calf being almost as hard as wood; no pulsation could be felt either in the leg or in the anterior or posterior tibial arteries.

It was evident that an artery had been broken; probably only recently by sloughing, and I decided to open up the leg and remove the clotted blood, and if possible, find the bleeding vessels and ligate them in the wounds. It was quite apparent that the high pressure from the hemorrhage within the leg had to be relieved.

Under ether narcosis I made a long incision over the front of the leg so as to include the wound of exit. As soon as the fascia was cut out burst a stream of blood several feet high. On account of the excessive hemorrhage I was compelled to tighten the rubber bandage which had been placed around the thigh just above the knee.

I had only one physician assisting me and he was giving the ether, and on account of this lack of assistance, decided to ligate the femoral artery in Scarpa's triangle, and trust to antiseptic treatment of the wounds as I had done in Case 1. The artery was ligated and a substantial protective dressing applied.

I was quite surprised a moment later when I began to clear out the wound in the leg to find already present evidences of intense infection. The wound in the leg was carefully cleansed with an antiseptic solution, while the rubber bandage yet remained tight to prevent absorption; thorough drainage was instituted, and the whole wound gently packed with iodoform gauze.

In the afternoon the temperature was 104, and during the night following there was a distinct chill. During the ten days following the temperature varied from 102 to 106; but the patient's appetite and strength kept up remarkably well; the temperature of the leg remained good, and there was no indication at any time of general gangrene. The tibialis anticus, however, became gangrenous and was removed to the tendon.

On the eighth day the dressing was removed from the ligation wound and the healing was found to be firm.

It was evident that the great mass of infected leg must be gotten rid of, and on the tenth day after the femoral artery was ligated, I amputated the thigh just above the knee. The parts were very vascular and everything bled, necessitating the use, as estimated by one of the assistants, of between fifty and sixty ligatures.

The amputation wound healed under one dressing; the temperature, immediately before the operation was 104½, and an hour afterwards was 99, and never rose above that point again. It was a typical case of sapremia.

On the eleventh day after the operation the patient was discharged with all wounds healed.

The judge before whom he was to be tried thinking he had suffered sufficient punishment dismissed him also.

In connection with the above cases a few remarks may be of interest. It would appear, perhaps, more surgical to open up such gunshot wounds and ligate the bleeding vessels near the points of injury, and if there were injured or severed nerves, they should be united. If the injury has been so severe as to make this procedure impossible an amputation should be done if the patient has sufficient strength.

In Case 1, of the foregoing report, the operation of ligating the subclavian artery was a life-saving one, and all such operations should be resorted to when necessary.

In Case 2 the operation was one of expediency, and also done with the hope that the leg might be saved. The ligation of the bleeding vessels in the wound might have saved the leg, but I doubt it; and the man came out of his injury with the leg only a little shorter than if the amputation had been done as a primary measure.

A few words in reference to the ligation of arteries: In healthy arteries and aseptic wounds it is a matter of no great consequence whether we use silk, catgut, kangaroo tendon, or any other form of ligature, so long as the ligatures are sterile. It would appear, also, in such arteries as the femoral and its branches, the brachial and its branches, and the smaller branches of the carotid—so long as the arteries are healthy—of little importance as to whether the middle coat of the artery is ruptured under the ligature or not. In the large arteries near the heart, in cases of aneurism and where the arterial walls have undergone degeneration, the ligatures should be large and smooth, not too easily absorbed, and applied in such way as to close the lumen of the vessel, and not to injure any of its walls. Silk floss, kangaroo tendon, and catgut, are very suitable ligatures for most ligations of the arteries. By soaking for a few days, the animal ligatures in a ten per cent. solution of formaldehyd, we may boil them at each operation just as we have been boiling silk thread for years. This greatly simplifies the question of animal ligatures, and puts the means of perfect sterilization at the ready command of us all.

I wish here to thank Drs. Trent, senior and junior, and Heflin, all of Roanoke, Ala., for assistance in Case No. 1, and Drs. Rea and Grady, of La Fayette, Ala., for assistance in Case No. 2.

